# High Mannose-Binding Antiviral Lectin PFL from *Pseudomonas fluorescens* Pf0-1 Promotes Cell Death of Gastric Cancer Cell MKN28 via Interaction with α2-Integrin

**DOI:** 10.1371/journal.pone.0045922

**Published:** 2012-09-20

**Authors:** Yuichiro Sato, Kinjiro Morimoto, Takanori Kubo, Kazuyoshi Yanagihara, Toshio Seyama

**Affiliations:** 1 Department of Medical Pharmacy, Faculty of Pharmacy, Yasuda Women's University, Hiroshima, Japan; 2 Department of Life Sciences, Faculty of Pharmacy, Yasuda Women's University, Hiroshima, Japan; Universidade de São Paulo, Brazil

## Abstract

Novel anti-HIV lectin family which shows a strict binding specificity for high mannose glycans has been found in lower organisms. The bacterial orthologue has been identified in the genome of *Pseudomonas fluorescens* Pf0-1 and the gene coding a putative lectin was cloned, expressed in *Escherichia coli* and purified by one step gel filtration. Glycan array screening of the recombinant lectin, termed PFL, has revealed that PFL preferentially recognizes high mannose glycans with α1-3 Man that was highly exposed at the D2 position. In contrast, masking of this α1-3 Man with α1-2 Man dramatically impaired lectin-carbohydrate interactions. Reducing terminal disaccharide, GlcNAc-GlcNAc of high mannose glycans was also essential for PFL-binding. PFL showed a potent anti-influenza virus activity by inhibiting the virus entry into cells at doses of low nanomolar concentration. At micromolar concentration or higher, PFL showed a cytotoxicity accompanying loss of the cell adhesion against human gastric cancer MKN28 cells. The cell surface molecule to which PFL bound was co-precipitated with biotin-labeled PFL and identified as integrin α2 by peptide mass fingerprinting using MALDI-TOF mass spectrometry. Intriguingly, upon treatment with exogenous PFL, integrin α2 on the cell surface underwent rapid internalization to the cytoplasm and accumulated to perinuclear region, together with the bound PFL. The resulting loss of cell adherence would trigger a signaling pathway that induced anoikis-like cell death. These events were effectively inhibited by pretreatment of PFL with mannnan, indicating the involvement of high mannose glycans on PFL-induced cell death that was triggered by PFL-integrin α2 interactions.

## Introduction

High mannose-binding lectins that target specific glycans on a virus surface are promising potential viral-inactivating agents that could be used for the prevention and control of virus infections [1], [2]. Numerous lectins that specifically recognize high mannose glycans are found from various taxonomy including bacteria, algae, plants and animals, and some of which have been shown to exhibit anti-HIV activity [3]. We have recently found a novel anti-HIV lectin family distributed in lower organisms including bacteria, cyanobacteria and marine algae [4]. They exhibit common characteristics, such as sequence multiplication of highly conserved N-terminal domain and exclusive high mannose oligosaccharide recognitions. Some lectins in this family such as cyanobacterial OAA from *Oscillatoria agardhii* [4] and red algal ESA-2 from *Eucheuma serra* [5] have shown to inhibit the HIV entry into the host cells with EC_50_s of low nanomolar range by directly binding to envelope gp120. Furthermore, a red algal lectin KAA-2 from *Kappaphycus alvarezii*, which also belongs to this family inhibits infection of various influenza virus strains with EC_50_s of low nanomolar levels in a strain-independent manner, through the recognition of high mannose oligosaccharide on the viral envelope glycoprotein hemagglutinin (HA) [6].

Besides the potent antiviral activity, ESA-2 shows various biological activities such as mitogenic activity for mouse and human lymphocytes and *in vitro* growth inhibition of tumor cells [7], [8]. Although biological properties of this lectin family are becoming apparent, the properties of bacterial orthologues from *Pseudomonas fluorescens* Pf0-1 and *Herpetosiphon aurantiacus* still remain to be clarified.

In the present study, we have cloned the orthologue lectin gene from *P. fluorescens* Pf0-1 and the coded lectin protein (PFL) was expressed in *Escherichia coli*. Functional PFL was successfully purified in high yield and characterized in terms of its biological activities such as antiviral and anti-tumor activity. As predicted from intense structural similarity of PFL with other members of this lectin family, PFL exhibited exclusive specificity for high mannose oligosaccharide and potent antiviral activity against influenza viruses. Furthermore, PFL induced anoikis-like cell death of gastric cancer cell MKN28 via interaction with cell surface integrin α2.

## Materials and Methods

### Materials

Stab culture of *P. fluorescens* Pf0-1 was generously provided by Dr. Mark W. Silby (University of Massachusetts Dartmouth, USA). Influenza viruses and Madin-Darby canine kidney (MDCK) cells were generously provided by Dr. T. Sakaguchi (Hiroshima University, Japan): The influenza viruses were grown in the chorioallantoic fluid of 10-day-old chicken eggs. MDCK cells were grown in Dulbecco's modified Eagle medium (DMEM) supplemented with 10% fetal bovine serum and penicillin-streptomycin. A stomach cancer cell line, MKN28 was kindly provided by Prof. Suzuki (Fukushima Medical University, Fukushima, Japan). The cell line was maintained in RPMI-1640 medium (GIBCO, Grand Island, NY) supplemented with 10% fetal bovine serum (FBS, GIBCO), 100 IU/mL penicillin G sodium, and 100 mg/mL streptomycin sulfate. Another stomach cancer cell line, GCIY, was purchased from RIKEN CELL BANK (Ibaraki, Japan) and maintained in the same way as described above. Primary normal human hepatocyte cell (ACBRI 3716) was purchased from DS Pharma Biomedical (Osaka, Japan) and maintained in CS-C medium kit R (DS Pharma Biomedical).

### Hemagglutination Assay

Hemagglutination assay was performed using a 2% (v/v) suspension of trypsin-treated rabbit erythrocytes as described previously [9]. Briefly, rabbit native erythrocyte suspension was treated with 0.5% trypsin in saline and the mixture incubated at 37°C for 60 min. After washing with saline, 2% trypsin-treated erythrocyte suspension was prepared in saline. Hemagglutination activity was expressed as a titer, the reciprocal of the highest 2-fold dilution exhibiting positive hemagglutination.

### Gene cloning and expression of lectin PFL

Genomic DNA from *P. fluorescens* Pf0-1 was used as a template for gene cloning of PFL gene. At first, a oligonucleotide primer set, 5′-GGCAGGTCTCCCGAAACTTCAAG-3′ and 5′-AGTCGAACGCCTGAACCTGTTGA-3′, which hybridized with upstream and downstream of the PFL coding region, respectively, were used, and the PCR was performed using Prime STAR DNA polymerase (TAKARA). Using the amplified PCR fragment as a template, the subsequent PCR was performed with a forward primer, 5′-CACCATGTCTAAATACGCAGTGGCA-3′, which had CACC additional sequence to ATG start codon of the PFL gene, and a reverse primer, 5′-TTACTCTATCTGCCCACGGAAG-3′ (TTA; corresponding to stop codon of the RFL gene). The amplified fragments were ligated into pET101/D-TOPO expression vector. The recombinant plasmid was transformed in *Escherichia coli* TOP10 cells (Invitrogen). The obtained recombinant clones were confirmed to be a correct construct by DNA sequencing. The functional clones were transformed in *Escherichia coli* BL21 Star (DE3) cells for inducible expression of the PFL gene. IPTG with a final concentration of 0.8 mM was added into the transformed culture to induce the PFL expression. After 6 h incubation at 37°C, the cells were harvested by centrifugation at 8000 rpm for 20 min.

### Purification of lectin PFL

The harvested bacterial cells were resuspended in 20 mM phosphate buffer (pH 7.0) containing 0.15 M NaCl (PBS) and were disrupted by sonication. After centrifugation at 8000 rpm for 20 min, an aliquot of the supernatant was subjected to Superose 12 column (GE Healthcare) equilibrated with PBS. The column was eluted with PBS at a flow rate of 0.8 ml/min by an isocratic mode. The eluate was monitored by absorption at 280 nm and examined for hemagglutination activity, and the active fractions were pooled.

### Molecular Weight Determination of PFL

The molecular weight of PFL was determined by MALDI-TOF MS analysis with an Autoflex mass spectrometer (Bruker, Japan) after the calibration with a peptide mass standard kit (Bruker, Japan) using sinapinic acid as a matrix.

### DNA sequence analysis

Nucleotide sequences were determined by the dideoxy chain terminator method using a BigDye Terminator version 3.1 Cycle Sequencing kit (Applied Biosystems). DNA sequencing was performed using an ABI 310 Genetic Analyzer (Applied Biosystems).

### Glycan array analysis

PFL was labeled with Alexa Fluor 488 according to the manufacturer's instructions (Invitrogen, Eugene, OR). Glycan binding specificity of PFL was determined by the printed glycan microarrays (version 5.0) according to the standard procedure of CoreH of the Consortium for Functional Glycomics (CFG) (http://www.functionalglycomics.org/glycomics/publicdata/primaryscreen.jsp). The average relative fluorescence in replicates of six was calculated by averaging four values after removing the highest and lowest values to eliminate some of the false hits with very high or low points. The error bars represent the standard error of the mean (SEM), and %CV is the coefficient of variation (S.D./mean) calculated as %.

### Anti-Influenza Activity of PFL

*In vitro* anti-influenza activity of PFL was determined by the neutral red (NR) dye uptake assay. Various concentrations of PFL were prepared with DMEM containing 10 μg/ml trypsin in a 96-well microplate. To each well, virus was added as a multiplicity of infection of approximately 0.001 infectious particles per cell. After incubating at 37°C for 48 h, 100 μl of NR dye (150 μg/ml in DMEM) was added and further incubated for 2 h. NR dye incorporated into the cells was extracted by the addition of 100 μl of 1% acetic acid/50% ethanol. The wells were measured at 540 nm with a microplate reader (1420 multilabel counter, PerkinElmer, MA, USA) as a factor of surviving from the virus cytopathic effect.

### Immunofluorescence Microscopy

Immunofluorescence staining was performed to visualize the entry inhibition of influenza virus by PFL. Briefly, MDCK cells grown on cover glass were infected with A/Udorn/72 at a multiplicity of infection of approximately 0.001 infectious particles per cell, in the presence or absence of 200 nM PFL in DMEM containing 10 μg/ml trypsin. After 24 h post infection, the infected cells were fixed with 80% acetone for 5 min. Following washing with PBS, the cells were incubated with mouse monoclonal anti- HA antibody (HyTest, Turku, Finland) at 37°C for 1 h. After washing with PBS, the cells were incubated with fluorescein isothiocyanate (FITC)-conjugated goat anti-mouse IgG antibody (Anticorps Secondaires, Compiègne, France) at 37°C for 1 h. After further PBS washing, the cells were mounted using Vectashield with 4′,6-diamidino-2-phenylindole (DAPI) (Vector Laboratories, Burlingame, CA) and were observed under a fluorescence-microscope (OLYMPUS BX51, Olympus, Japan).

### ELISA assay

Direct interaction of PFL with viral envelope glycoprotein HA was assayed using an enzyme-linked immnosorbent assay (ELISA). PFL (5 μg/ml) in carbonate buffer (pH 9.6) was coated on 96 well ELISA plates (BD Biosciences, Bedford, MA). The wells were washed three times with PBS containing 0.1% Tween20 (PBST) and blocked with 3% skim milk at 37°C for 1 h. After washing with PBST, varying concentrations of influenza HA vaccine preparation (Astellas, Tokyo, Japan) were added to each well and incubated at 37°C for 1 h. After washing with PBST, the wells were incubated with mouse anti-HA monoclonal antibody (HyTest) at 37°C for 1 h followed by incubation with horse-radish peroxidase (HRP)-conjugated goat anti-mouse IgG antibody (GE Healthcare, UK) at 37°C for 1 h. Subsequently 3,3′,5,5′-tetramethylbenzidine (TMB) substrate (Sigma-Aldrich, Saint-Louis, MI) was added. The reaction was stopped using TMB stop reagent (Sigma-Aldrich) and absorbance at 450 nm was measured using a microplate reader (1420 multilabel counter, PerkinElmer).

### Tumor cell proliferation by MTS assay

Cell proliferation was quantified by a conventional MTS assay using CellTiter 96 cell proliferation assay (Promega, Madison, WI). Cells seeded on 96-well microplate were incubated for 72 h with various concentrations of PFL in appropriate medium with 10% FBS. The cells were then incubated with 20 μl of MTS reagent for 1 h at 37°C and measured with a microplate reader (1420 multilabel counter, PerkinElmer) at 490 nm. The effect of yeast mannan on cytotoxity of PFL was determined by incubating the cells with 5 μM PFL in the presence of various concentrations of yeast mannan in RPMI 1640 with 10% FBS for 72 h, and cell viability was measured as described above.

### Isolation of PFL binding molecule(s) on MKN28 cell

PFL was labeled with biotin using biotin labeling kit (Dojindo molecular technologies, Japan) according to the manufacture's instructions. To confluent MKN28 cells on cover slips, biotinylated-PFL (200 μg) was added and incubated for 2 h at room temperature. After washing with cold PBS, the cells were scraped off and lysed in 800 μl of RIPA buffer (50 mM Tris-HCl, pH 7.6, 150 mM NaCl, 1% Nonidet P40, 0.5% Sodium deoxycholate, protease inhibitor cocktail, 0.1% SDS). Subsequently, 100 μl of biotin-capture avidin beads (Adar Biotech, Israel) was added to the cell lysate and incubated overnight at 4°C with gentle agitation. The beads were washed three times with 50 mM Tris-HCl, pH 7.5. Captured proteins on beads were eluted with 50 μl of SDS-PAGE sample buffer (62.5 mM Tris, pH 6.8, 2% SDS, 10% glycerol, 1% mercaptoethanol, 0.003% bromphenol blue) for 15 min at 90°C and subjected to SDS-PAGE. The protein band specific for PFL treatment fraction was analyzed by MALDI-TOF MS after in-gel digestion with trypsin. Briefly, the CBB stained protein bands were cut out and destained with 25 mM ammonium bicarbonate containing 50% acetonitrile. The reductive alkylation was performed with 50 mM TCEP (Tris [2-carboxyethyl] phosphine) in 25 mM ammonium bicarbonate, followed by incubation with 50 mM iodoacetoamide for 1 h. After dehydration with acetonitrile, the protein in the gel was digested with 10 μl of TPCK-trypsin (100 μg/ml) in 50 mM ammonium bicarbonate. The digestion was purified with Zip tip (Millipore, Japan) and spotted onto a MALDI target. One μl of matrix solution (α-cyano-4-hydroxycinnapic acid matrix [Bruker, Japan] in acetone-ethanol [1∶2]) was then added to the spot. MALDI-TOF MS analysis was carried out by an Autoflex mass spectrometer (Bruker, Japan) after the calibration with a peptide mass standard kit (Bruker, Japan). The peptide mass finger printing data was searched by the Mascot software (Matrix Science, Japan).

### Cellular localization of PFL and integrin α2

MKN28 cells were cultured on coverslips in a 6-well plate. The cells growing on coverslips were treated with 30 μg/ml Alexa488 conjugated-PFL in RPMI 1640 and incubated for different time periods. After washing with PBS, the cells were fixed with 80% acetone for 5 min. Following washing with PBS, the cells were incubated with mouse monoclonal anti-integrin α2/CD49b antibody (R&D Systems, MN) at 37°C for 1 h. After washing with PBS, the cells were incubated with Alexa568-conjugated goat anti-mouse IgG antibody (Life technologies, Japan) at 37°C for 1 h. After further PBS washing, the cells were mounted using Vectashield with DAPI (Vector Laboratories) and were observed under confocal laser scanning microscope (IX70; Olympus, Japan). By employing the other gastric cancer cell line GCIY and normal human hepatocyte cells ACBRI 3716, cellular localizations of integrin α2 and Alexa488-PFL were examined in the same way as described above, and observed under a fluorescence microscope (OLYMPUS BX51). The effect of yeast mannan on cellular localization of PFL and integrin α2 was evaluated as follows. Confluent MKN28 cells grown on coverslips in a 6-well plate were treated with 20 μg/ml of Alexa488-PFL in RPMI 1640 in the presence or absence of 700 μg/ml yeast mannan for 4 h. The cells were fixed, visualized using anti-integrin α2/CD49b antibody as described above, and observed under a fluorescence microscope (OLYMPUS BX51). Effect of various lectins on cellular localization of integrin α2 was examined in a similar way, by incubating the MKN28 cells with 20 μg/ml of each lectin in RPMI 1640 for 4 h.

## Results

### Molecular cloning, expression and purification of PFL

**Figure 1:**
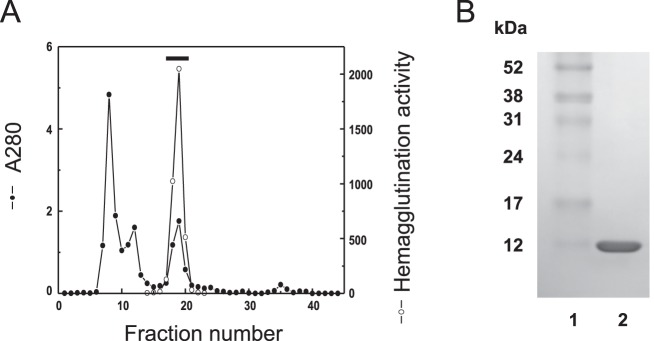
Purification of PFL. (**A**) Gel filtration on a Superose 12 column. An aliquot of cell lysate from *E. coli* expressing PFL was subjected to Superose 12 column equilibrated with 0.15 M NaCl in 20 mM phosphate buffer (PBS). The column was eluted with PBS at a flow rate of 0.8 ml/min by an isocratic mode. The eluate was monitored by absorption at 280 nm and examined for hemagglutination activity, and the active fractions denoted by bar were pooled. (**B**) SDS-PAGE of purified PFL. The purified PFL was applied on 10% PAGE under reducing conditions. Lane 1, molecular weight standards; Lane 2, purified PFL.

We have previously found the genome of *P. fluorescens* Pf0-1 contains a possible homologue of anti-HIV lectin family that was recently found in lower organisms including bacteria, cyanobacteria and marine algae [4]. Based on the nucleotide sequence of hypothetical lectin of *P. fluorescens* Pf0-1 on the database, primer sets were designed to amplify the lectin gene. Putative lectin gene was successfully cloned by directional TOPO cloning system, and the coding protein was heterologously expressed in *E. coli* BL21 (DE3). The expressed lectin protein was purified to homogeneity by a single step gel filtration on Superose 12 column (Fig. 1A). The active peak with hemagglutination activity gave a single protein band of 13 kDa on SDS-PAGE (Fig. 1B). Finally, from 1 litter *E. coli* culture, high yield of purified lectin (240 mg) was obtained. The purified lectin was named PFL and used for further examination. The molecular mass of PFL (13883.7) determined by MALDI-TOF MS was in agreement with the calculated mass (13881.1) from the deduced amino acid sequence and that estimated value by the mobility on SDS-PAGE.

**Figure 2:**
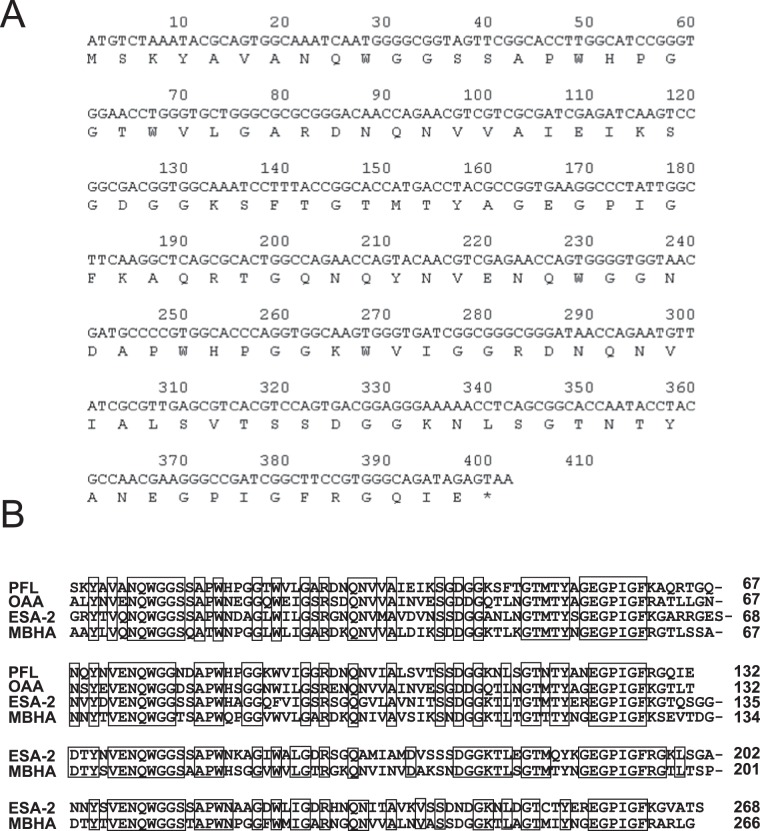
Nucleotide and deduced amino acid sequences of PFL. (**A**) The stop codon TAA is shown as an asterisk. Each number represents the position of nucleotide. The sequence was completely same as shown in GenBank databases under accession no. ABA72252. (**B**) Alignment of amino acid sequences of PFL and other homologous lectins of anti-HIV lectin family. Cyanobacterial lectin; OAA from *Oscillatoria agardhii* (SwissProt accession no. P84330), Red algal lectin; ESA-2 from *Eucheuma serra* (SwissProt accession no. P84331), Bacterial lectin; MBHA from *Myxococcus xanthus* (GenBank accession no. M13831). Identical amino acids among four lectins are boxed.

The deduced amino acid sequence of PFL harbored two homologous domains, each consisting of the N- and C-terminal halves with 62% sequence identity between them. PFL exhibited high sequence homology to cyanobacterial lectin OAA from *Oscillatoria agardhii*, red algal lectin ESA-2 from *Eucheuma serra*, and bacterial lectin MBHA from *Myxococcus xanthus* (Fig. 2B), which constitute a novel anti-HIV lectin family. The molecular mass of PFL and OAA were similar, 13883.7 and 13924.1, respectively, and both lectins were composed of 132 amino acids. Both PFL and OAA have a common property in its sequence duplication but OAA displays higher degree of internal sequence identity with 75% between the two repeated domains. In contrast, ESA-2 and MBHA are composed of four tandemly repeated homologous domains of 67 amino acids. The degrees of similarity of OAA, ESA-2 and MBHA with PFL in their N-terminal portions (each 132 residues) of the amino acid sequences were 62.1, 61.4, and 62.1% for identical amino acids, respectively.

### Carbohydrate-binding specificity of PFL

**Figure 3:**
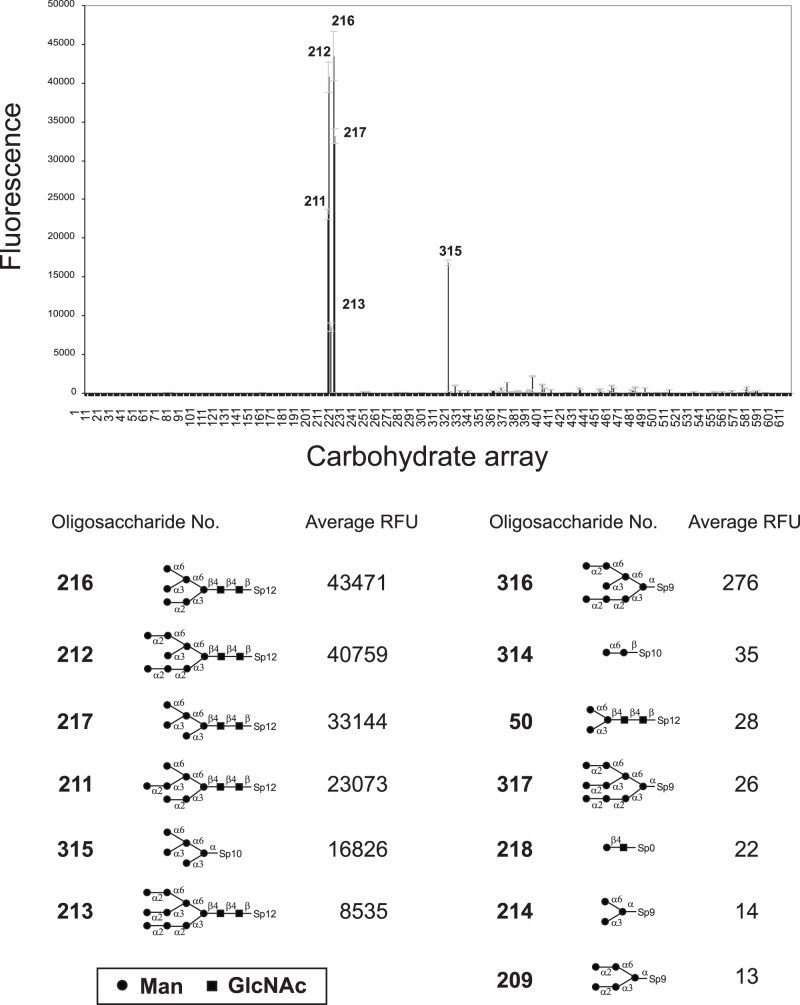
Glycan array analysis of PFL. Glycan binding specificity of PFL was determined by the printed glycan microarrays v5.0 according to the standard procedure of CoreH of the Consortium for Functional Glycomics. Error bars represent mean ± standard deviation. RFU value represents the relative fluorescence units. The glycan array data was obtained with 10 μg/ml of Alexa488-PFL.

To determine carbohydrate-binding specificity of PFL, glycan array analysis was performed at the Consortium for Functional Glycomics using printed array version 5.0. Of the 611 kinds of oligosaccharides tested, PFL (10 μg/ml) showed exclusive specificity for high mannose type glycans as shown in Fig. 3. The entire list of oligosaccharide tested and the results can be found online at (http://www.functionalglycomics.org/glycomics/publicdata/primaryscreen.jsp).

PFL strongly bound to M6 glycan (**216**) and M8 glycan (**212**), both of which have an exposed α1-3 Man on the D2 arm, with the similar levels of high RFU values, 43471 and 40759, respectively. In contrast, masking of this α1-3 Man with α1-2 Man dramatically impaired the interaction between PFL and the glycans. This was most evident by a comparison of M6 glycan (**216**) and M7 glycan (**211**), where the additional α1-2 Man at D2 arm decreased the binding potency approximately to 53%. Similarly, binding potency of PFL to M9 glycan (**213**) was decreased (RFU = 8535) compared with its counterpart M8 glycan (**212**) lacking D2 terminal α1-2 Man, showing only 21% of the potency. Interestingly, removal of the reducing terminal disaccharide, GlcNAc-GlcNAc of high mannose glycans much drastically diminished PFL-binding. For instance, the PFL binding potency was almost completely abolished to the glycans **316** and **317** having no GlcNAc-GlcNAc sequence whereas those counterpart glycans **212** and **213**, respectively, exhibited much higher potency. The importance of terminal GlcNAc-GlcNAc was further confirmed by the comparison of glycans **217** and **315**, although the extent of impairment of PFL-binding was limited. This lectin was devoid of monosaccharide-binding including mannose. Moreover, PFL did not interact with Man α1-6 Man (**314**) and mannotriose (**214**), which are constituents of the branched portion of high mannose glycans. Pentasaccharide core of N-glycan (**50**) was not recognized by PFL but its fucosylated counterpart (**485**) displayed weak interaction (RFU = 746). These oligosaccharide binding profiles of PFL were closely similar to those of OAA, ESA-2 and KAA-2 that belong to a novel anti-HIV lectin family in lower organisms [4]–[6].

### Anti-Influenza Virus Activity of PFL

**Figure 4:**
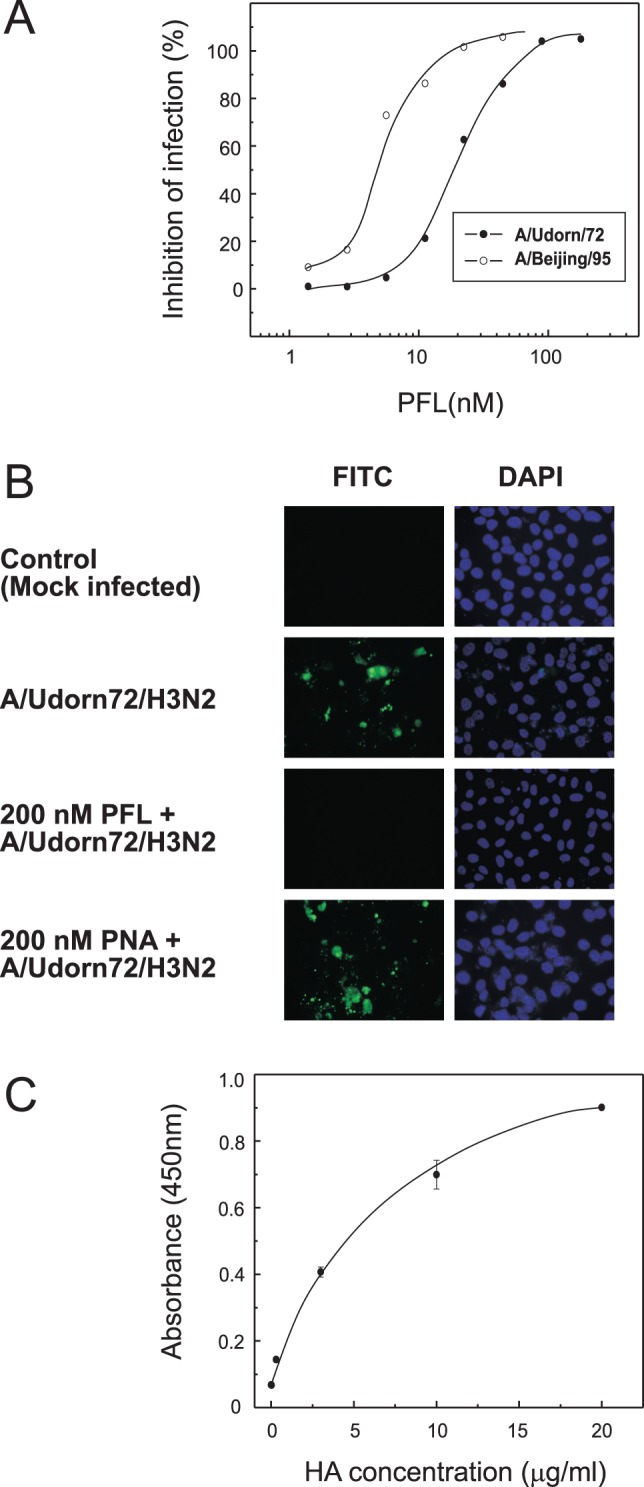
Anti-influenza virus activity of PFL. (**A**) Anti-influenza activity of PFL in MDCK cells infected with two influenza virus strains. Cell viability following incubation for 48 h with influenza viruses in the presence of varying concentrations of PFL was assayed using the NR dye uptake assay. Percent inhibition of infection is expressed as the average value of duplicate assays. Open circles, A/Beijing/262/95 (H1N1); closed circles, A/Udorn/72 (H3N2). (**B**) Inhibition of influenza virus entry into MDCK cells by PFL. MDCK cells were infected with A/Udorn/72 (H3N2) in the presence or absence of 200 nM PFL. After 24-h infection, the cells were fixed with 80% acetone for 5 min. Viral antigens in the infected cells were detected by the specific antibody against viral envelope glycoprotein (HA) and FITC-conjugated 2nd antibody under a fluorescence microscope. Nuclei within the cells were stained with DAPI (200× magnification). (**C**) Direct binding of PFL to viral envelope glycoprotein HA. Interaction between PFL and viral HA was assayed by an ELISA. PFL was immobilized onto a 96-wells plate and incubated with various concentration of an influenza vaccine which contained HA from A/California/7/09 (H1N1), A/Victoria/210/09 (H3N2), and B/Brisbane/60/08. The wells were incubated with mouse anti-HA monoclonal antibody for 1 h at 37°C followed by with HRP-conjugated 2nd antibody for 1 h at 37°C. The HRP substrate, TMB, was then added to the wells and the absorbance at 450 nm was measured. The figure shows results of one experiment that was replicated at least twice with similar results.

Anti-influenza virus activity of PFL was evaluated with two influenza virus strains, A/Udorn/72 (H3N2) and A/Beijing/262/95 (H1N1) by the NR dye uptake assay. PFL effectively inhibited cytopathic effect caused by both influenza virus strains, with EC_50_s of 19.4±1.5 nM, and 4.5±0.4 nM, respectively (Fig. 4A). To confirm that PFL inhibited the initial step of influenza virus entry into the cells, distribution of viral antigens in the infected cells was observed in the presence or absence of PFL using immunofluorescence microscopy. Fig. 4B shows distribution of the viral antigen after 24 h post infection with A/Udorn/72 detected by specific anti-hemagglutinin antibody. PFL efficiently inhibited influenza virus entry into the cells whereas the viruses were able to penetrate and replicate in the host cells in the absence of PFL. A galactose specific lectin PNA from *Arachis hypogaea* failed to inhibit virus entry into the cells. These results suggest the presence of high mannose glycans on the virus surface at the position critical for virus entry into cells. To test whether PFL would directly bind to viral envelope glycoprotein HA, an ELISA assay was performed with commercially available vaccine preparation which contain HA from A/California/7/09 (H1N1), A/Victoria/210/09 (H3N2), and B/Brisbane/60/08 as a major component. As demonstrated in Fig. 4C, dose-dependent binding of HA to PFL was observed.

### PFL induced cell death of human gastric cancer cell MKN28

**Figure 5:**
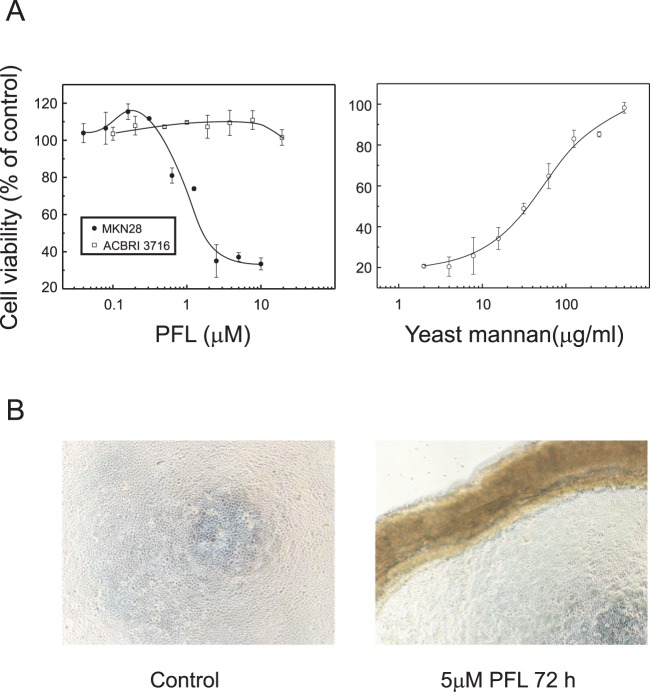
Cytotoxicity of PFL on human gastric cancer MKN28 cells. (**A**) Cytotoxicity of PFL on MKN28 cells (left panel; black circles) and the effect of yeast mannan on cytotoxicity of PFL (right panel). Human MKN28 cells were incubated with varying concentrations of PFL for 72 h. At the end of incubation, the cell survival rate was determined by MTS methods. The cytotoxicity is expressed as the percentage of cell survival rate compared with the control. The figure shows results of one experiment that was replicated at least twice with similar results. Effect of yeast mannan on PFL cytotoxicity was determined after incubating the MKN28 cells with 5 μM PFL in the presence of various concentrations of yeast mannan for 72 h. As a reference, the effect of PFL on normal human hepatocyte cells (ACBRI 3716) is shown (left panel; white squares) (**B**) Microscopic image of control (left) and PFL-treated (right) MKN28 cells. The cells were incubated for 72 h in the presence or absence of 5 μM PFL and were observed under a light microscopy.

*In vitro* anti-tumor effect of PFL on gastric cancer cell MKN28 was evaluated by conventional MTS assay. As shown in Fig. 5A (left panel), PFL showed a dose-dependent effect on the proliferation of MKN28 cell. At 72 h post PFL-treatment, cell viability was significantly decreased at doses of 0.5 μM or higher. In contrast, at the low doses of 0.1–0.3 μM, cell proliferation was slightly stimulated. The PFL-induced cell death of MKN28 cells was accompanied by a loss of cell adhesion to the bottom of culture plate, as shown in Fig. 5B where everted cluster of cells were observed as brownish lines. The PFL-induced cell death was effectively inhibited in the presence of yeast mannan, a glycoprotein bearing high mannose oligosaccharides (Fig. 5A, right panel). This indicates the high mannose glycans on MKN28 cells are involve in PFL-induced cell signaling that ultimately leading to cell death. In contrast, normal human hepatocyte cells (ACBRI 3716) were relatively resistant to PFL treatment compared with MKN28 cells (Fig. 5A, left panel).

### Isolation of PFL-binding molecule(s) on human gastric cancer cell MKN28

**Figure 6:**
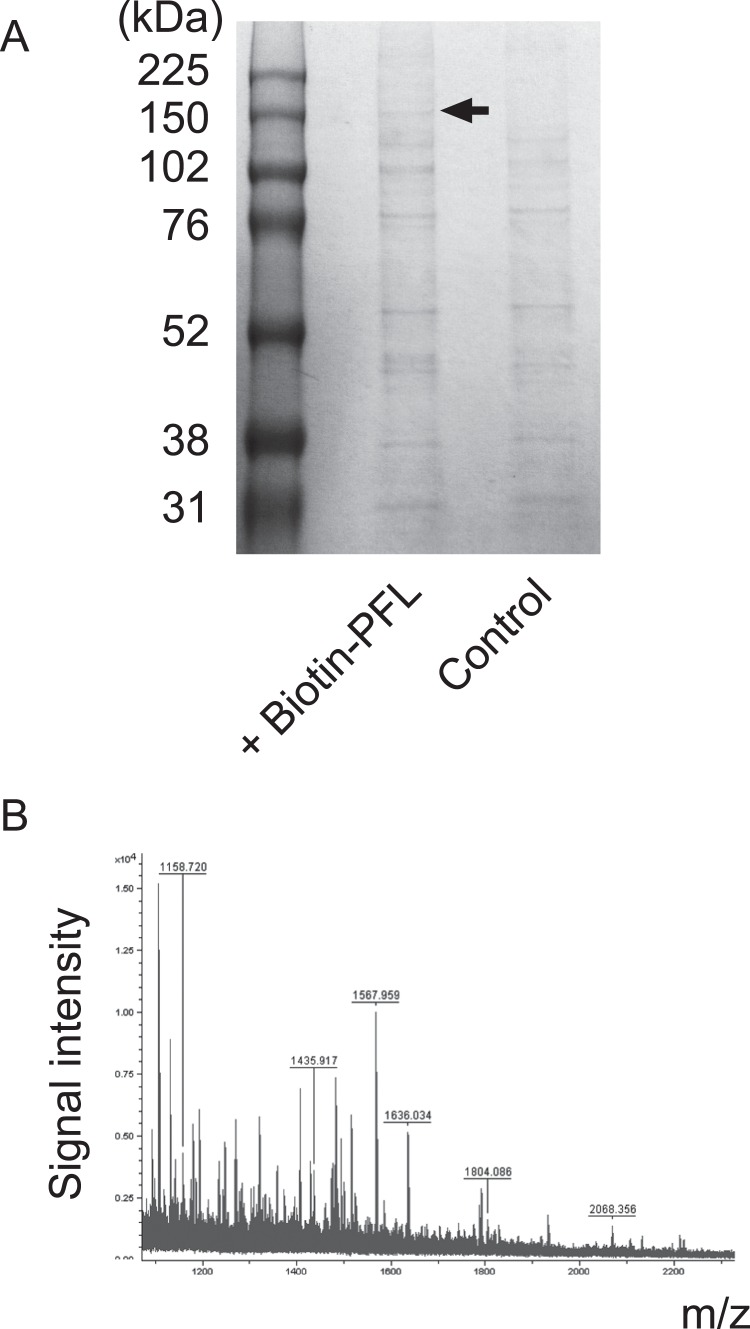
Identification of PFL-binding molecule(s) on MKN28 cell. MKN28 cells were treated with biotin-PFL (200 μg) and incubated for 2 h at room temperature. After lysing the cell with RIPA buffer containing 0.1% SDS, proteins that bound to biotin-PFL were precipitated with avidin beads. Captured proteins on beads were eluted with SDS-PAGE sample buffer by incubating for 15 min at 90°C and subjected to SDS-PAGE (**A**). The 150 kDa protein band specifically eluted from PFL fraction was analyzed by MALDI-TOF MS after in-gel digestion with trypsin. The peptide mass finger printing data (**B**) was searched by the Mascot software.

To address the molecular mechanism by which PFL induces cell death of MKN28 cell, cell surface molecule(s) to which PFL bound were investigated. Biotinylated PFL was incubated for 2 h with MKN28 cells and the cells were lysed with RIPA buffer containing 0.1% SDS. The cell surface molecule(s) to which biotin-PFL bound were co-precipitated with avidin-coated beads. The proteins trapped on beads were subsequently eluted with SDS-PAGE sample buffer and analysed on SDS-PAGE (Fig. 6A). Although several non-specific bands were detected, we observed a 150 kDa band which specifically detected in PFL treated fraction. This band was further subjected to in-gel digestion of trypsin followed by MALDI-TOF mass spectrometric analysis. The obtained peptide mass fingerprinting data (Fig. 6B) were searched for database and the protein was identified as integrin α2, with the probability based scores of 57 (p<0.05).

### Cellular localization of exogenously added PFL and integrin α2

**Figure 7:**
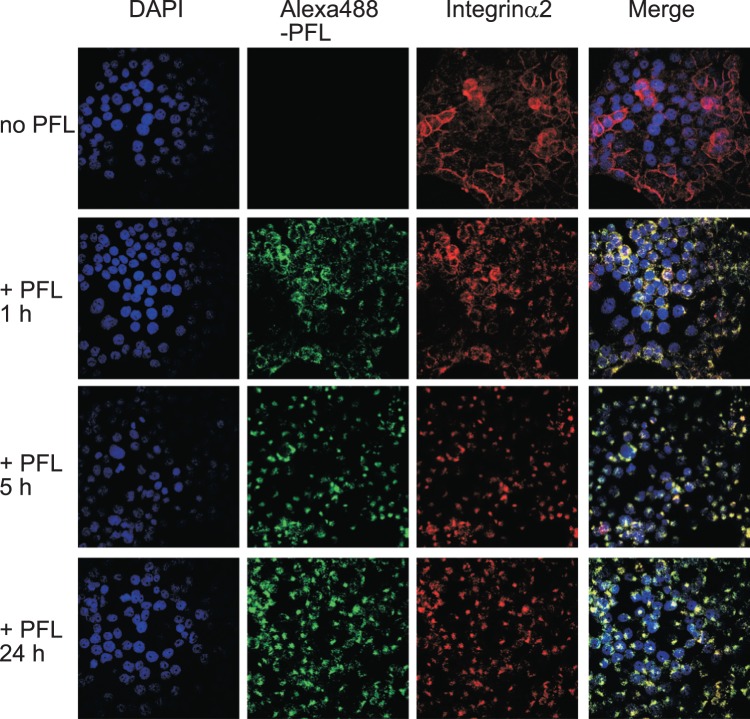
Cellular distribution of integrin α2 in MKN28 cells upon treatment with exogenous PFL. The cellular localizations of integrin α2 and Alexa488-PFL were assessed by immunofluorescence using a confocal microscope. MKN 28 cells were incubated with 30 μg/ml Alexa488-PFL in RPMI for different time periods. Subsequently, the cells were fixed with 80% acetone and visualized with mouse monoclonal anti-integrin α2/CD49b antibody and Alexa568-conjugated goat anti-mouse IgG antibody and were observed under a confocal laser scanning microscope. Nuclei within the cells were stained with DAPI. Colocalization of Alexa-488 PFL and integrin α2 is shown as yellow signals (right lane).

**Figure 8:**
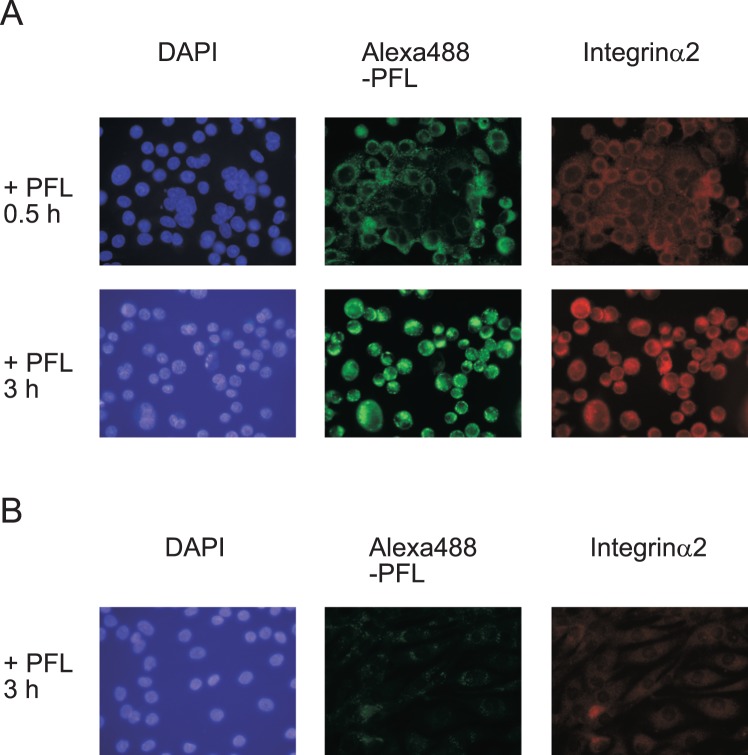
Distributions of integrin α2 upon treatment with PFL in GCIY or ACBRI 3716 cells. The cellular localizations of integrin α2 and Alexa488-PFL were examined in the similar way with MKN28 cells. GCIY cells (**A**) or ACBRI 3716 cells (**B**) were incubated with 30 μg/ml Alexa488-PFL in RPMI 1640 or CS-C medium for indicated time periods and visualized with mouse monoclonal anti-integrin α2/CD49b antibody and Alexa568-conjugated goat anti-mouse IgG antibody. The images were observed under a fluorescence microscope.

The behaviors of PFL itself and integrin α2 upon treatment with PFL were examined using a confocal fluorescent microscope. In this experiment, fluorescent-labeled PFL (Alexa488-PFL) was used to track PFL localization. An Alexa488-PFL was incubated with MKN28 cells for 1, 5 and 24 h at doses of 30 μg/ml. Integrin α2 were detected with the anti-integrin α2/CD49b monoclonal antibody followed by Alexa568-conjugated 2nd antibody. Interestingly, Alexa488-PFL bound to the cell surface and initiated internalization to cytoplasm within 1 h (Fig. 7). Integrin α2 which was predominantly localized on cell surface at steady state also underwent internalization, and importantly, the localization of integrin α2 coincided well with PFL. After 5 h incubation, both PFL and integrin α2 were co-localized and accumulated in perinuclear region and further incubation basically did not alter the location of both proteins. It is therefore likely that once PFL bound to integrin α2, both proteins have never been recycled back to the cell surface. Rapid intracellular trafficking of PFL-integrin α2 complex occurred even on collagen I coated cover slips (data not shown). Similarly, redistribution of integrin α2 upon treatment with PFL was observed in another gastric cancer cell line, GCIY, but not in normal human hepatocyte cells, ACBRI 3716 (Fig. 8). In ACBRI 3716 cells, internalization of PFL was not observed possibly by the lesser expression of integrin α2 on cell surface.

### Involvement of high mannose glycans on PFL-integrin α2 interaction

**Figure 9:**
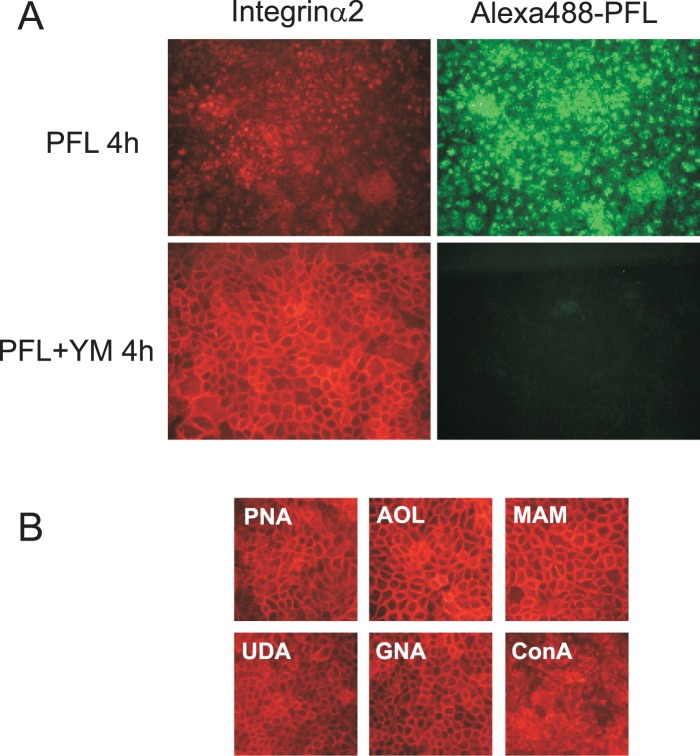
Involvement of high mannose glycans on PFL-integrin α2 interaction. (**A**) Effect of yeast mannan (YM) on PFL-induced internalization of integrin α2 was determined by immunofluorescence using a fluorescence microscope. MKN 28 cells were incubated with 20 μg/ml Alexa488-PFL in RPMI for 4 h in the presence or absence of 700 μg/ml yeast mannan (YM). The cells were fixed with 80% acetone and visualized with mouse monoclonal anti-integrin α2/CD49b antibody followed by with Alexa568-conjugated 2nd antibody. (**B**) Effect of various lectins on cell surface integrin α2. MKN 28 cells were incubated with 20 μg/ml of various lectins with different specificity in RPMI for 4 h. The cells were visualized using an anti-integrin α2/CD49b antibody as above.

To test whether cellular redistribution of integrin α2 caused by PFL may arise from a specific interaction of PFL with high mannose glycans on integrin α2 molecule, we have evaluated the effect of yeast mannan on PFL-induced integrin α2 internalization using MKN28 cells. In the absence of yeast mannan, both proteins underwent significant internalization (Fig. 9A, upper panels). In contrast, in the presence of yeast mannan, PFL failed to bind to cell surface, and the subsequent intracellular trafficking of PFL was completely abolished (Fig. 9A, lower right). Agreeing to this observation, integrin α2 did not change its localization in the presence of yeast mannan (Fig. 9A, lower left). These results clearly indicate that PFL bound to integrin α2 through the recognition of high mannose glycans on integrin α2. To further confirm the requirement of high mannose glycans for integrin α2 redistribution, we have tested the effect of various lectins on cellular localization of integrin α2. As shown in Fig. 9B, other lectins with different specificities such as galactose-binding PNA from *Arachis hypogaea*, fucose-binding AOL from *Aspergillus oryzae*, sialic acid-binding MAM from *Maackia amurensis*, D-GlcNAc-binding UDA from *Urtica dioica* did not affect the location of integrin α2. Interestingly, even high mannose binding lectins such as a monocot mannose (Man)-binding lectin, GNA from *Galanthus nivalis* did not show any significant change on integrin α2. In contrast, high mannose binding legume lectin ConcanavalinA (ConA) distorted the arrangement of integrin α2 like PFL did.

## Discussion

In this study, we have evaluated the biological activity of the novel bacterial lectin PFL from *P. fluorescens* Pf0-1 that belongs to recent found anti-HIV lectin family in lower organisms. Occurrence of increasing drug-resistant influenza virus strains as well as emerging highly pathogenic virus strains such as avian H5N1 led us to explore PFL as a novel anti-influenza agent. PFL showed potent anti-influenza virus activity and the mechanism by which PFL inhibited virus replication was inhibition of the initial step of virus entry into cells. It is most likely that PFL exerted anti-influenza activity by selectively binding to high mannose glycans on viral envelope HA, as demonstrated in ELISA assay [9]. In fact, site specific occurrence of high mannose oligosaccharide has shown at the region of HA near the receptor binding site [10].

To explore anti-tumor effect of PFL, we employed human gastric cancer cell line MKN28 which is originated from a moderately differentiated intestinal type tumor. The red algal lectin ESA-2 which belongs to the same lectin family with PFL exhibited anti-tumor effect both *in vitro* and *in vivo* [7], [8]. ESA-2 is a protein derived from the edible seaweed and expected to exert anti-tumor effect on gastrointestinal tract cancer by oral administration. It is notable that PFL promoted cell death of MKN28 cell, exclusively through a recognition of high mannose glycans on integrin α2 molecule, which predominantly located on cell surface at steady state. Heterodimeric integrins act not only as anchorage molecule to attach cells to an appropriate extracellular matrix (ECM) but also as sensors of the ECM environment [11]. Depending on the state of cell attachment on ECM, integrins activate either anti-apoptotic or apoptotic signaling [12]. When cells are failed to bind to proper ECM, those cells undergo integrin-mediated cell death or “anoikis” defined as an apoptosis that results from the loss of cell adhesion to the ECM, or inappropriate adhesion [13]. Upon binding of PFL to surface integrin α2, both proteins have caused rapid internalization to the cytoplasm. As observed in the confocal microscopic images, PFL and integrin α2 formed clustered complex and co-localized at perinuclear region and never have been recycled back to the cell surface. Therefore, PFL-induced cell death accompanying cell detachment would be strongly associated with the down-regulation of integrin α2 at cell surface. In this context, it has been reported that clustering of α2β1 integrin with antibodies and echovirus 1 (EV1) causes redistribution of α2 integrin to perinuclear multivesicular bodies and become susceptible to calpain-dependent degradation but not proteasomal and autophagosomal degradation [14]. In contrast, ConA, which also binds to high mannose glycans, shows cytotoxicity to hepatoma cells by inducing autophagy. ConA is also internalized to the cytoplasm but preferentially localized onto mitochondria by the unknown mechanism [15]. Our data, however, suggest that integrin molecules might also be involved in the process of ConA internalization. Recently, endogenous lectin galectin-1 (Gal-1) has shown to stimulate anoikis via functional interaction with the fibronectin receptor α5β1 integrin [16]. Mutation studies of *N*-glycosylation site on α5β1 integrin have demonstrated the importance of *N*-glycosylation on hetero-dimer formation, cell spreading, actin cytoskeltal formation and proper folding of the subunit [17]. Moreover, numerous studies have shown glycosylation status of integrin have pivotal roles in many aspects of tumor cell adhesion and migration as well as cancer metastasis [17]–[20]. PFL-induced cell death with concomitant internalization of PFL into the cells and cellular redistribution of α2 integrin was effectively inhibited in the presence of yeast mannan, a glycoprotein with high mannose glycans. Other lectins with different specificities such as galactose-binding PNA, fucose-binding AOL, sialic acid-binding MAM and D-GlcNAc-binding UDA failed to change locations of α2 integrin. Of note, even high mannose binding lectins such as GNA did not affect the α2 integrin distribution. These results implicated the presence of certain high mannose glycan(s) on α2 integrin which specifically recognized by PFL.

As revealed by glycan array screening, PFL specifically recognized branched oligomannosides of high mannose glycans with preference for those having the exposed α1-3 Man from the α1-6 arm of core trimannoside (D2 arm). To oligosaccharide having α1-2 Man attached at the α1-3 Man in D2 arm, binding potency of PFL was significantly reduced. It is most plausible that steric hindrance of α1-2 Man is account for disruption of the interaction. Recently, overall architecture of OAA, which showed similar amino acid sequence and oligosaccharide binding profile with PFL, has revealed as a novel fold with unique ten stranded β-barrel-like structure [21]. Two symmetrical carbohydrate binding sites with distinct affinity have shown to locate on the protein. NMR titration study has suggested OAA mainly recognizes either of the two Man α1-6 Man units imbedded within the branched oligomannoside [21]. It has been suggested that the binding cleft seems too short to accommodate the reducing terminal GlcNAc residue(s) of high mannose glycans. Given the intense similarity of amino acid sequences and molecular weights between OAA and PFL, these lectins might have the same folding. However, our data indicated the portion of the core GlcNAc residue(s) appeared to be essential for interaction with PFL, in comparison of the binding potency between the oligosaccharide having the GlcNAc residues and its counterpart lacking the GlcNAc residues. This was most evident by a comparison of the binding potencies of PFL with M8 oligosaccharide **212** (RFU  = 40759) and **316** (RFU  = 276). Thus, the reducing terminal GlcNAc residue(s) is likely to serve as a subsite in the recognition of high mannose glycans by PFL, as we have previously shown in other lectins in this family, such as OAA, ESA-2 and KAA-2 [4]–[6].

It has been reported that both subunits of integrin α3β1 contained bi-, tri- and tetra-antennary complex type oligosaccharides as a major component, but high mannose type oligosaccharide was a minor [22]. Although it is still unclear how integrin initiates its internalization with PFL but not with other lectins of different specificity, we assume that high mannose glycan(s) are located in the region crucial for the function of integrin or integrin-mediated signaling. For PFL-induced cell death, functional interaction of PFL with cell surface integrin α2 through high mannose glycan was a prerequisite but the following mechanistic aspects leading death signaling need to be elucidated in the future. Integrin α2 has shown to mediate selective metastasis to the liver [23] and thus *in vivo* effect of PFL is also of interest for investigation.

The physiological roles of PFL as well as other lectins in this family also remain to be clarified. The originated bacteria *P. fluorescens* inhabits soil, water and plant surface environments. The fact that PFL protein is coded only on the genome of *P. fluorescens* Pf0-1 strain but not on the other sequenced strain such as Pf-5 was not surprising because *P. fluorescens* genome are highly diverse [24]. In turn, *P. fluorescens* Pf-5 contains lectin-like bacteriocin which consists of tandem monocot mannose-binding lectin domain [25]. The strong antibiotic activity of ESA against fish pathogenic bacteria [26] suggests that PFL also serves as a bacteriocin against agricultural pathogens under physiological conditions. The origin of these lectin proteins is still controversial but it is possible that ancestral molecule of marine algal lectins might derive from bacterial symbionts. In fact, fluorescent *Pseudomonas* has been found in the rhizoid of marine algae [27].

In the present study, we have demonstrated the interesting biological activities such as antiviral and anti-tumor activity of a novel bacterial lectin PFL from *P. fluorescens* Pf0-1. This protein would be useful not only as a potential candidate for antiviral or anti-tumor reagent but as a tool for biochemical and biomedical research. However, careful attention should be paid to control desirable PFL concentration depending on the purpose of its application, since the biological activity exerted by lectins sometimes show reverse dose responses [28].
